# Cognitive Dysfunction of Chikungunya Virus Infection in Older Adults

**DOI:** 10.3389/fpsyt.2022.823218

**Published:** 2022-04-05

**Authors:** Vanessa Giffoni M. N. P. Peixoto, Julianna P. Azevedo, Kleber G. Luz, Katie M. Almondes

**Affiliations:** ^1^Post-graduation Program in Psychobiology, Universidade Federal do Rio Grande do Norte, Natal, Brazil; ^2^Department of Clinical Medicine, Universidade Federal do Rio Grande do Norte, Natal, Brazil; ^3^Department of Psychology, Universidade Federal do Rio Grande do Norte, Natal, Brazil; ^4^Department of Infectious Diseases, Institute of Tropical Medicine, Universidade Federal do Rio Grande do Norte, Natal, Brazil; ^5^Technical Advisory Group for Arbovirus, World Health Organization, Geneva, Switzerland

**Keywords:** chikungunya virus, older adults, elderly, cognition, dementia, cognitive decline, cognitive dysfunction, elders

## Abstract

**Background:**

Chikungunya fever is a disabling articular disease caused by chikungunya virus (CHIKV). In the past decade it has affected millions of people across America, Africa, Asia, and Europe, turning this infection into a public health concern. The acute phase of chikungunya infection is usually self-limiting, characterized by severe arthralgia, fever, chills, myalgia, headache, and rash. CHIKV neurovirulence is evident and seems to be higher among elders. Considering their susceptibility to cognitive decline and dementia, the aim of our study was to investigate whether CHIKV infection might cause long-term cognitive impairment in aged people.

**Methods:**

A cross-sectional study was conducted with volunteers aged from 60 to 90 who had been affected by chikungunya and also with healthy controls. A structured questionnaire was used to record demographic and clinical data, functional status, and depression. Global cognitive function was assessed through MoCA. A comprehensive neuropsychological battery was performed to assess specific cognitive functions.

**Results:**

Subjective memory complaints were present in 70% of subjects with previous chikungunya. This group had a poorer performance in MoCA (*p* = 0.000) and specific cognitive tests: Semantic (*p* = 0.05) and Phonemic Verbal Fluency (*p* = 0.003), 5-Digit (choice, reading, counting and alternance, *p* = 0.003, *p* = 0.014, *p* = 0.021, and *p* = 0.021, respectively), Stroop test (time, errors and interference, *p* = 0.000, *p* = 0.027 and *p* = 0.015, respectively), and RAVLT (word total session *p* = 0.05). These tests reflect performance on general executive functions, cognitive flexibility, inhibitory control, processing speed, semantic memory and episodic memory.

**Conclusion:**

Our data suggest that CHIKV infection may cause long-term cognitive decline in aged people and might be a risk factor for future dementia in this population.

## Introduction

Chikungunya fever is a disabling articular disease caused by the chikungunya virus (CHIKV), a single-stranded RNA arbovirus that belongs to the *Togaviridae* family. Its transmission to humans is mainly mediated by female bytes of *Aedes* mosquitoes, the same vectors for dengue, Zika, and yellow fever viruses (1), besides vertical transmission. CHIKV was first identified in the 1950s in Tanzania, spreading to other countries in Africa and Asia, and later to Europe and America. In the past 8 years, an alarming increase in countries with autochthonous transmission has affected millions of people across America, Africa, Asia, and Europe (2), turning this infection into a public health concern worldwide (3).

The acute phase of chikungunya infection is characterized by severe arthralgia or arthritis, fever, chills, myalgia, headache, and maculopapular rash (1). The disease is usually self-limiting, but some patients may progress to a chronic stage, in which persistent and disabling joint pain may continue for months or years (4–6). In 2015, the World Health Organization (WHO) proposed a consensus regarding the clinical presentations of CHIKV infection, comprising the “acute clinical” presentation—only classical symptoms are present, “atypical” cases, when additional organs are affected, and “severe cases,” if there is at least one organ failure that might be life threatening or require hospitalization (7).

Atypical and severe presentations are more prevalent in older populations (8–10), possibly reflecting the high prevalence of underlying chronic conditions and the aging-related changes in the immune system (9). CHIKV neurovirulence seems to be higher among older people, and the severely ill (11) and neurological complications are probably the commonest forms of severe presentations, accounting for approximately 25% of atypical and 60% of severe cases (12, 13). The term “neuro-chikungunya” describes the range of neurological involvement in chikungunya fever and accounts for controversial direct viral central nervous system (CNS) infection and autoimmune-mediated mechanisms. The first one would be more common in newborns and older people, justified by flaws in the innate and adaptive immune response, and it would explain the cases of encephalopathies. Experiments with murines demonstrated that CHIKV is capable of infecting neurons, astrocytes, and oligodendrocytes, causing apoptosis in the first 2. It is also capable of crossing the choroid plexus and infecting rat meninges and ependyma with altered interferon signaling. On the other hand, the autoimmune pattern generally presents itself later under the forms of optic neuritis, Guillain–Barré, and encephalomyelitis. In these cases, CHIKV would act in a molecular mimicry mechanism, unleashing autoantibodies against the myelin sheath of Schwann cells or against gangliosides of Ranvier's nodules, resulting in demyelination, blockage of nerve impulse conduction, and axonal damage (13).

A retrospective cohort telephone survey identified subjective complaints of memory and attention in participants with previous history of chikungunya, although lacking objective cognitive analysis (14). Although previous studies have shown controversial neurocognitive outcomes in perinatally infected children (15–17), there is insufficient data concerning long-standing investigation of cognitive symptoms in adults after chikungunya disease. A range of infectious diseases cause important nervous system involvement, including viruses (for example, HIV, herpes simplex virus, Japanese encephalitis virus, cytomegalovirus, arboviruses, and varicella zoster virus), parasites (e.g., neurocysticercosis, malaria, schistosomiasis), bacteria (e.g., syphilis, tuberculosis, bacterial meningitis), and fungi (e.g., cryptococcus). Millions of children and adults, especially those living in low- and middle-income countries, suffer from neurological, cognitive, or mental health problems as a result of these infections. Nonetheless, precise estimates of morbidity and neurological pathogenesis of some infections are still unexplored (18). There is a clear rationale for neurological sequelae after serious CNS infections, such as encephalitis and meningitis, but it is still unknown whether some neurotropic viruses might cause long-term cognitive problems even in the absence of neurological symptoms during the acute presentation.

Cognition concerns a range of mental processes related to the acquisition, storage, manipulation, and retrieval of information (19). It is crucial for several daily tasks across lifetime and also a condition for autonomy along the aging process. Cognition might be studied as distinct domains or brain circuits and neuromodulators; hence, cognitive assessment is a useful tool to test for cognitive impairment, either with a screening test or through a formal neuropsychological battery for detailed investigation. Several conditions may result in cognitive impairment, such as neurodegenerative disorders, chronic psychiatric problems and acquired brain damage due to vascular damage, trauma, hypoxia, metabolic disturbances, and infection. The aging process is threatened by dementia and mild cognitive impairment, not only by the cognitive complaints but also through behavioral and functional decline.

Considering CHIKV neurotropism and the known relationship between viral infections and cognitive deficits, our hypothesis is that long-term cognitive complaints may arise when elders—more susceptible to cognitive decline—are affected by chikungunya disease, even in the absence of neurologic symptoms in the acute phase. The aim of our study is to investigate whether CHIKV infection might cause cognitive impairment in aged people.

## Materials and Methods

### Study Design and Participants

A cross-sectional study was conducted with volunteers between October and December 2019, in Natal, a Brazilian city located on the northeastern coast. Eligible study participants were those aged from 60 to 90 years old with higher than 4 years of formal education. Social networks disseminated a call for volunteers who had been affected by chikungunya disease in the same year. Subjects with previous cognitive decline, uncontrolled psychiatric conditions, or recent stroke, heart attack, or cardiac arrest were excluded. Participants with clinical manifestations of “neuro-chikungunya” in the acute disease were also excluded from the analysis. Healthy controls were enrolled by the same inclusion and exclusion criteria. A total of 135 participants were assessed through a comprehensive interview that lasted approximately 2 h and took place at the Institute of Tropical Medicine, Universidade Federal do Rio Grande do Norte. Volunteers who had been affected by CHIKV were requested to present a serologic confirmation of the infection. A certified geriatrician and neuropsychologist completed the assessments with the aid of trained research assistants.

### Instruments

A structured questionnaire was used to record data on demographics, subjective memory complaints, and sleep conditions. Pfeffer's Functional Activities Questionnaire (FAQ) (20) and the Geriatric Depression Scale (GDS) (21) checked for functional status and depressive symptoms, respectively. Global cognitive function was assessed through the Brazilian version of Montreal Cognitive Assessment (MoCA) (22, 23) using a cutoff point of 25 points for cognitive impairment. To represent the most relevant metric of MoCA for clinical practice, the performances were binary classified based on the cutoff as normal, whenever the score from the test was at least 25, or deficient, otherwise. Correction for years of education was performed for the global MoCA score (+ 1 point in the case of <12 years of education). To better analyze each cognitive domain, a set of subtests was selected to create a neuropsychological battery specific for this population. All tests are validated in our population and are used internationally. The battery included Boston Naming Test (short version) (24) for language; Stroop Test (25) for selective attention, inhibitory control and processing speed; Rey Auditory Verbal Learning Test (RAVLT) (26) for episodic memory and verbal learning; Semantical and Phonemical Verbal Fluency Test (27) for language, executive functions, and semantic memory; Five Digit Test (28) for processing speed and executive functions; Frontal Assessment Battery (29) for motor control and executive functions; Psychological Battery Tests of Attention (PBTA) (30) for selective, sustained, divided and alternate attention; Stick Design Test (31) for visuoconstructive skills. Raw scores on all neuropsychological tests were converted to age- and education-adjusted standard scores using published norms developed for the Brazilian population. The MoCA and neuropsychological tests results are reported as mean [±standard deviation (SD)]. For neuropsychological assessment, z-scores were established according to the means and standard deviations for each variable and adjusted by age and education where applicable; impaired performance on a test was defined as −1.5 SD below the mean of the respective normative group.

### Statistical Analysis

The data collected were entered into MS Excel and further analyzed using the R software. Means and standard deviations were calculated for clinical and socio-demographic characteristics of the sample. According to Shapiro–Wilk test, normally distributed continuous variables were analyzed by *t* test and non-normally distributed ones by Mann–Whitney non-parametric test. Chi-square test was used for group comparisons on nominal variables, although differences in impairment (< -1.5 SD) between groups was limited by the small number of controls. The probability of having the performance on MoCA classified as deficient was modeled as a function of the occurrence (or not) of previous chikungunya infection by means of logistic regression. For controlling such an effect, demographic variables were included in the statistical model as predictors if the sample provided evidence substantial enough to suggest its influence on the response or interaction with other explanatory variables. The selection of predictors was supported by exploratory analysis, and it was confirmed by (backward) stepwise automatic procedure and manual exploration of related models. To avoid sparsity of the data and to mitigate imbalance (in terms of sample sample), some categorical explanatory variables had their levels pooled into fewer categories. At last, the statistical model was validated based on the evaluation of corresponding diagnostic measures, residues, and confirmatory analysis. The statistical significance of the model's parameters was conditioned to a significance level of α = 0.05 in a Wald test.

### Ethics

This study was approved by the local Research Ethics Committee of the University Hospital Onofre Lopes, Natal, Brazil, member of the National Research Ethics Committee under the process number 20420119.0.0000.5292. Written informed consent was obtained from all participants prior to conducting the study. The information collected was kept confidential.

## Results

A total of 121 older adult subjects out of 135 were eligible for the study, of which 95 were affected by chikungunya disease (CHIK) in 2019 (mean of 6 months later), and 26 were healthy controls (HC) (Supplementary Material). Half of the study participants were between the age groups of 60 and 68 years, with a mean age of 68.91 years (standard deviation: 5.92, range: 60–87). Two-thirds of participants had subjective memory complaints, and 68% of those were worried about this issue, although only 44% reported that the memory complaint started in the last year. Depressive symptoms were present in 20% of the sample. Insomnia was reported in 36% of the subjects, representing the most prevalent sleep complaint in both groups. Of those, only 27% addressed the last year as the onset of the complaint. Memory complaints were statistically more prevalent among the CHIK group (*p* = 0.001), whereas depressive symptoms and sleep complaints were not (*p* = 0.231 and *p* = 0,812, respectively). All subjects were functionally unimpaired according to the Pfeffer scale (Pfeffer score <5). Table 1 summarizes the clinical and demographic characteristics of the study population. Raw neuropsychological test results and inferential statistics are described in Table 2, as well as the number of impaired results in both groups according to scores below −1.5 SD (standard deviation) for each test.

**Table 1:** Demographic and clinical characteristics by groups (CHIK and healthy controls).

	**CHIK**	**HC**	***x*** ^**2**^	***p***
	**(*n* = 95)**	**(*n* = 26)**		
**Age**—M ± SD	68.08 ± 5.70	68.24 ± 6.93		
**Sex—*****n*** **(%)**				
Female	66 (69.5)	19 (73.1)	0.127	0.722
Male	29 (30.5)	7 (26.9)		
**Marital status—*****n*** **(%)**				
Single	11 (11.6)	2 (7.7)		
Married	66 (69.4)	14 (53.8)		
Divorced	7 (7.4)	7 (26.9)		
Widow	11 (11.6)	3 (11.5)		
**Schooling—*****n*** **(%)**				
4–8 years	2 (2.1)	1 (3.8)		
9 years	6 (6.3)	0		
12 years	33 (34.7)	9 (34.6)		
University degree	43 (45.3)	13 (50)		
Postgraduation	11 (11.6)	3 (11.5)		
**Income—*****n*** **(%)**				
Lower than R$1.100	1 (1.1)	0		
R$ 1.000–1.600	5 (5.3)	0		
R$ 1.600 e 3.000	9 (9.5)	2 (7.7)		
R$ 3.000 e 5.000	14 (14.9)	3 (12.0)		
R$ 5.000 e 10.000	33 (35.1)	5 (20.0)		
R$ 10.000 e 23.000	27 (28.8)	13 (50)		
Higher than R$ 23.000	6 (6.3)	2 (7.7)		
**Occupation**—***n*** **(%)**				
Retired	72 (75.8)	11 (42.3)		
Working	20 (21)	14 (53.7)		
Unemployed	3 (3.2)	1 (3.8)		
**Memory complaints**—***n*** **(%)**				
No	25 (26.3)	16 (61.5)	11.305	**0.001**
Yes	70 (73.7)	10 (38.5)		
**Sleep complaints**—***n*** **(%)**				
No	56 (58.9)	16 (61.5)	0.057	0.812
Yes	39 (41.1)	10 (38.5)		
**Depressive symptoms**—***n*** **(%)**				
No	74 (77.9)	23 (88.8)	1.433	0.231
Yes	21 (22.1)	3 (11.5)		

*Values are expressed in mean results (M) ± standard deviation (SD) or number of subjects (n)/frequencies (%). Chi-square (x^2^) test for group differences, with significant results (p < 0.05) shown in bold*.

*CHIK, individuals with previous chikungunya; HC, healthy controls; R$, Brazilian Reais (currency)*.

*The Brazilian minimum wage is R$1,100.00*.

**Table 2:** Raw neuropsychological tests results and the respective impairment (tests results < -1.5 SD) by groups (CHIK and healthy controls).

**Neuropsychological tests**	**CHIK**	**HC**	**t or U**	***p***
	**(*n* = 95)**	**(*n* = 26)**	***x*** ^**2**^	
**MoCA test**—M ± SD	21.56 ± 3.26	24.69 ± 2.54	−4.51	**0.000**
Deficit (score <25) n (%)	79 (83.2%)	13 (50.0%)	12.316	**0.000**
**Stroop test—M** **±SD**				
Time 1st sheet	16.13 ± 3.67	14.10 ± 2.15	841.000	**0.013**
Errors 1st sheet	0.08 ± 0.37	0.00 ± 0.00	1,157.00	0.191
Time 2nd sheet	24.53 ± 6.60	20.58 ± 4.65	786.500	**0.006**
Errors 2nd sheet	0.05 ± 0.33	0.00 ± 0.00	1,196.000	0.361
Time 3rd sheet	38.18 ± 10.50	29.63 ± 8.12	662.000	**0.000**
Errors 3rd sheet	0.62 ± 1.34	0.07 ± 0.27	977.000	**0.027**
Interference	2.37 ± 0.52	2.09 ± 0.46	2.46	**0.015**
**Semantical verbal fluency test—M** **±SD**				
Animals	15.88 ± 4.70	18.50 ± 6.56	956.500	0.078
Score *Z*	1.17 ± 1.26	1.90 ± 1.70	924.000	**0.050**
**Phonemical verbal fluency test—M** **±SD**				
Words	33.00 ± 11.11	40.88 ± 13.88	838.000	**0.012**
Score *Z*	1.27 ± 1.38	2.25 ± 1.67	−3.032	**0.003**
**Stick design test—M** **±SD**				
Total Score	11.59 ± 0.70	11.26 ± 0.96	991.000	0.073
**Five digit test—M** **±SD**				
Reading	27.08 ± 9.04	24.19 ± 5.52	838.000	**0.014**
Counting	28.50 ± 6.26	25.73 ± 5.62	860.500	**0.021**
Choice	47.51 ± 10.53	41.11 ± 10.64	767.500	**0.003**
Inhibition	20.15 ± 8.46	17.38 ± 8.59	1.475	0.143
Flexibility	43.19 ± 17.67	38.11 ± 17.65	963.00	0.099
Alternance	70.22 ± 19.61	62.73 ± 20.48	859.00	**0.021**
Deficit alternance (< -1.5 SD) *n* (%)	24 (25.3)	5 (19.2)	0.408	0.523
**RAVLT—M** **±SD**				
Word total	34.57 ± 7.89	38.11 ± 8.67	−1.981	**0.050**
A7 List	6.07 ± 2.82	7.11 ± 3.32	1,024.00	0.205
Recognition	7.05 ± 4.80	7.28 ± 4.58	1,141.500	0.827
**PBA—M** **±SD**				
Concentrated attention	74.34 ± 19.01	78.80 ± 20.81	−1.038	0.301
Divided attention	34.95 ± 24.43	47.61 ± 24.88	−1.779	0.078
Alternated attention	56.95 ± 23.49	66.00 ± 27.32	−1.678	0.096
General attention	166.80 ± 56.76	188.26 ± 67.47	−1.639	0.104
**Frontal assessment battery**—M ± SD	15.60 ± 2.49	16.23 ± 1.96	1,059.500	0.259

*Values are expressed in mean results (M) ± standard deviation (SD) or number of subjects with scores < -1.5 SD (n)/frequencies (%). t-test (t) or Mann–Whitney (U) for comparisons between means and Chi-square (x^2^) test for group differences. Significant results (p < 0.05) shown in the 5th column in bold*.

*x^2^ analysis was limited by the small number of controls*.

*CHIK, individuals with previous chikungunya; HC, healthy controls; RAVLT, Rey Auditory Verbal Learning Test; PBA, Psychological Battery Tests of Attention*.

**Figure 1:**
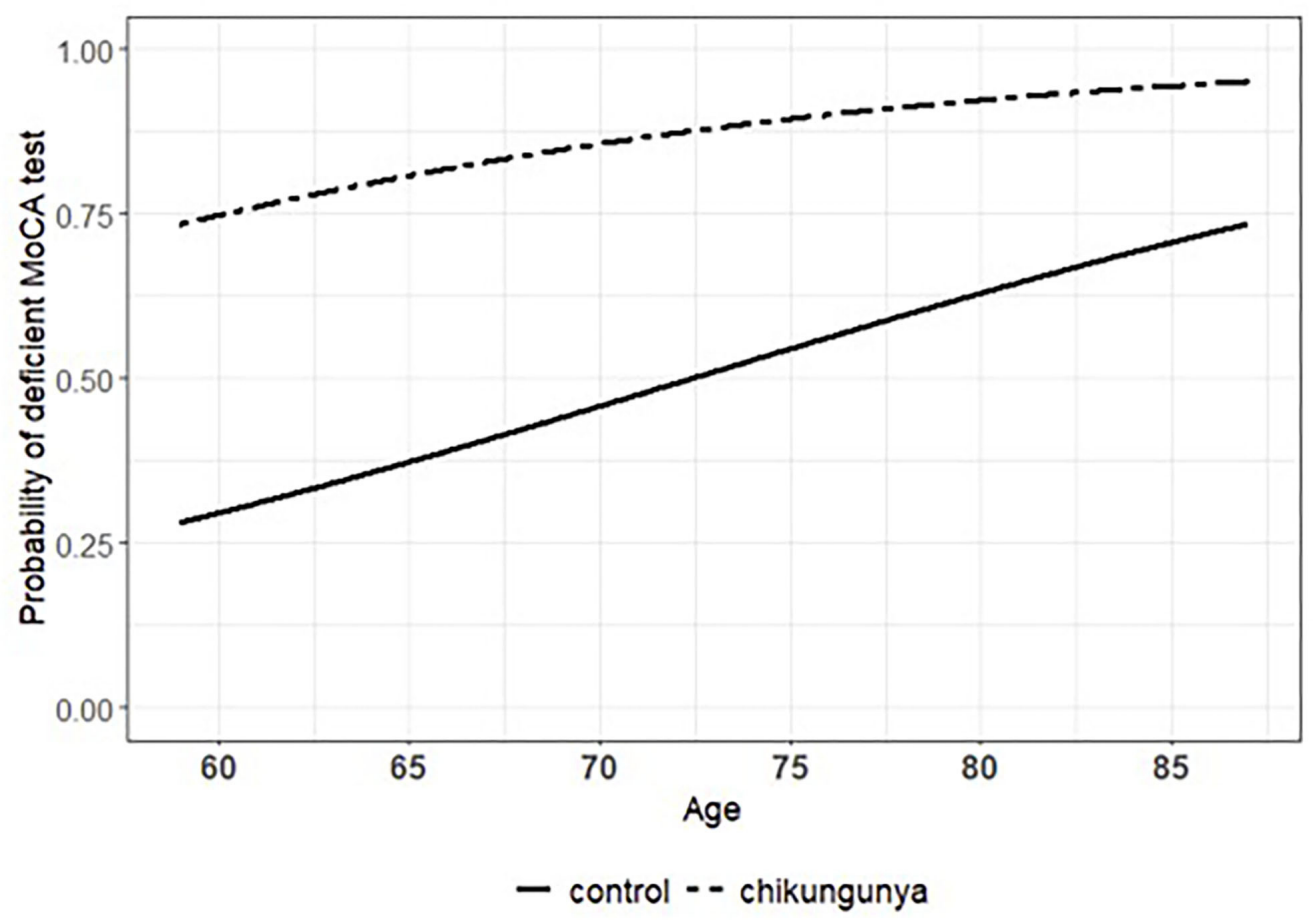
Estimated association (or effect) of chikungunya infection and participant's age on the probability of having the performance on the Montreal Cognitive Assessment (MoCA) test considered as impaired.

Comparing both groups, CHIKV (+) subjects had worse performance in MoCA than in HC (t_119_ = 4.51, *p* = 0,000), which shows evidence of cognitive decline among individuals with previous chikungunya. According to statistical modeling, the probability of presenting a deficient performance on the MoCA test could be explained by the previous occurrence (or not) of infection with chikungunya virus along with the participant's age (Table 3). In this context, while age was kept constant, the infection with chikungunya virus was associated with a 607, 29% increment in the odds of having the performance on MoCA considered as deficient compared with the healthy controls (odds ratio = 7.072). Accordingly, the statistical significance of such an association was confirmed under robust amount of evidence provided by the sample (Table 3; Wald test: coeff = 1.956, Std. Error = 0.494, *Z* = 3.956, *p* = 0.000). The effect of chikungunya infection on MoCA performance can be appreciated by taking the difference between the curves in Figure 1. In turn, the association between age and the classification of performance on the MoCA test did not reach statistical significance (Table 3; Wald test: coeff = 0.069, Std. Error = 0.043, *Z* = 1.614, *p* = 0.106). Nevertheless, according to diagnostic measures (Supplementary Figure 9) and confirmatory analysis (Supplementary Table 1), what could be considered at the first glance as fragility of evidence provided by the sample was actually the result of bias introduced by two unusually influential observations that hindered the true pattern of the data: the risk of impaired performance on MoCA test increased as the participant grew older. Under these circumstances, while keeping the chikungunya exposure constant, every 1-year increment in the participants' age was associated with a 7.242% increase in the odds of presenting deficient performance on the MoCA test (odds ratio = 1.072). Such an effect takes shape as the ascent of both curves in Figure 1.

**Table 3:** Estimated coefficients for the logistic regression that models the probability of deficient performance on the MoCA test as a function of chikungunya infection and the participant's age.

**Logistic regression (Wald test)**				
	**Estimate**	**Std. error**	***z***	***p***
(Intercept)	−5.062	2.974	−1.702	0.088
Chikungunya	1.956	0.494	3.956	**0.000**
Age	0.069	0.043	1.614	0.106

*Each coefficient is followed by the respective Wald test and statistics*.

*Significant results (p < 0.05) shown in the fifth column in bold*.

HC scored better in Phonemic Verbal Fluency Test (Z score *p* = 0.003) and Semantic Verbal Fluency Test—animal category (Z score *p* = 0.05) and also performed more efficiently in the Five Digit Test—categories reading (*p* = 0.014), counting (*p* = 0.021), choice (*p* = 0.003), and alternance (*p* = 0.021). In the Stroop test, HC completed the first, second, and third sheets faster than CHIKV (+) (*p* = 0.013, *p* = 0.006, and *p* = 0.000, respectively), recorded more accurate answers in the third sheet (*p* = 0.027) and controlled better for interference (*p* = 0.015). HC also had superior performance in the verbal and auditory learning test—word total session (*p* = 0.05), but there was no relevant difference in recognition (*p* = 0,827). These data suggest that the CHIK group presented evidence of impairments in general executive functions, cognitive flexibility, inhibitory control, mental processing speed, semantic memory, and episodic memory.

## Discussion

Our study suggests that patients with CHIKV infection experience long-term cognitive problems; we found very high rates of self-reported subjective cognitive complaints and also poorer performance in global and some specific cognitive tests in subjects previously affected by CHIKV.

Ninety-five individuals, who had had classic manifestations of chikungunya (without neurological symptoms or the need for hospitalization) in 2019, were analyzed. Evaluation of 26 healthy volunteers was performed to pair the sample. Intriguingly, in our study, reports of subjective memory complaints exceeded 70% in the group previously affected by chikungunya (CHIK). This prevalence is higher than what was informed by participants of the TELECHIK (14) survey, with 42% and 37% of memory and attention complaints, respectively.

The presence of subjective cognitive complaints refers to the self-perception of a decrease in cognitive performance, which may be validated or not by the objective confirmation (cognitive testing) of such a decrease (32). The term subjective cognitive decline is used to characterize cognitively unimpaired individuals with cognitive concerns, and its importance resides in a greater risk of progression to mild cognitive impairment (MCI) and dementia (33). Many individuals use terms such as “forgetfulness” or “memory problems” to translate the perception of change in other cognitive domains, such as, for example, executive function, language, attention, and mental processing speed. These complaints may arise from diverse clinical situations, for instance, regular aging, psychiatric diseases, metabolic and vascular diseases, drug abuse, among others (33). The perception of cognitive decline by the individual or informant is also central for the diagnosis of MCI and dementia, considering that in these situations, the proving of cognitive decline through testing is required. Finally, the distinction between the latter conditions is dependent on functional impairment, indispensable for diagnosing dementia (34, 35). In our study, all participants were functionally unimpaired, which means that no person with dementia was enrolled.

### Global Cognitive Testing

We found a difference in the MoCA average scores between the CHIK and control (HC) groups, indicating a lower global cognitive score in previously infected elders. In another analysis, when we compared normal or altered (MoCA <25) results between the groups, it was also confirmed that there are more people with declined global cognition in the CHIK group, even after controlling for background variables. We considered the cutoff of 25 in the MoCA test due to the considerable prevalence of subjects with high schooling in our sample (23), although a few individuals with lesser education may have affected mean scores even in the control group (23). The detailed analysis of the cognitive functions showed that the CHIK group has performed worse in tests that assess general executive functions, cognitive flexibility, inhibitory control, processing speed, as well as episodic and semantic memory.

### Executive Functioning and Viral Infections

Executive functions are understood as a set of mental processes in charge of the general cognitive control, including the orchestration of multiple basic cognitive functions (36). These are required in practically all aspects of daily life, such as developing ideas, planning actions, solving unexpected problems, resisting impulses, adapting to unexpected situations, thinking before making decisions, performing tasks simultaneously, and paying attention to stimuli in spite of distracting elements (37). According to the model proposed by Diamond (37), they are divided into working memory, inhibitory control, and cognitive flexibility; the two last components presented themselves as compromised in our sample.

Besides evaluating language skills, an unfavorable phonemic fluency, as demonstrated in CHIK subjects, is considered a marker of dysexecutive functioning once lack of initiative, perseveration, or difficulty in following rules might result in fluency impairment. In order to achieve a greater number of words starting with a specific letter, the subject must group words in subcategories and switch to the next category once the last one has been exhausted. If semantic fluency is dependent on semantic knowledge and is specially related to the temporal lobe, accessing subcategories requires cognitive flexibility and is mainly dependent on the frontal lobes (27, 36). Processing speed is another important requirement for a successful fluency output, influencing most cognitive tasks (37). Our study also demonstrated that elders with previous chikungunya had worse results in most sessions of the Stroop test (number of errors and time to complete the test), which is also a measure of processing speed, cognitive flexibility, and inhibitory control. Finally, the results presented in the Five Digit test confirm the impairment in interference control, processing speed, and focusing and swapping attention.

Interestingly, executive dysfunction is one of the main cognitive aspects of frontal-subcortical dementias (38), due to alterations in the frontal-subcortical circuits, specially within the dorsolateral prefrontal cortex and its subcortical connections with the caudate, globus pallidus, and thalamus (39). Disruption in these circuits may also explain the slowing of processing speed found in our sample.

Executive dysfunction and deficit in immediate visual memory were also found 1 year after infection by another arbovirus, the West Nile virus, even in patients that did not present encephalitis in the acute phase, which raised the hypothesis that this pathogen may damage frontal-subcortical structures in a subclinical way, with an evident long-term impairment (40). Another infection, which is known to harm executive functioning, is the human immunodeficiency virus (HIV), which also compromises attention, memory, processing speed, learning, and motor control. HIV may affect several brain regions, especially the basal ganglia, with consequent disruption of frontal-striatal circuits (41). Individuals suffering from chronic hepatitis C virus infection (HCV) also present cognitive dysfunctions in executive functions, verbal learning, memory, and attention, irrespective of the grading of liver fibrosis, probably due to a pattern of neuroinflammation, which compromises the basal ganglia and white matter (42). It is worth mentioning that these infections assume a chronic nature, whereas arbovirus infection is acute and self-limited, despite some studies pointing to the possibility of chronicity (43).

### Semantic and Episodic Memory

Human memory can be divided into implicit and explicit memory systems, the latter comprising semantic, episodic, and also working memory (44). Whereas semantic memory refers to the knowledge acquired on things and their functions, facts, concepts, words, and their meanings (44), episodic memory relates to autobiography, registering temporally dated episodes or events and consciously recalling them (44, 45). Anterior and inferior temporal lobe cortex are responsible for semantic memory, which is prematurely and intensely affected in semantic dementia, and also in herpes simplex virus (HSV) encephalitis (46, 47) and later in Alzheimer's disease (AD) (44). The medial temporal lobe/hippocampus is in charge of processes related to episodic memory (encoding, consolidation, and retrieval), strongly influenced by the prefrontal cortex, which organizes the input and output of information (48). In our study, subjects with previous chikungunya performed worst in semantical fluency (suggestive of semantic memory and/or language impairment) and also in the auditory and verbal learning test, reflecting a learning/memory deficit. On the other hand, both groups had similar scores in the recognition task, which suggests that subjects might have not forgotten the learned information but struggled to access them without a cue. This pattern is compatible with dysfunction in the frontostriatothalamocortical circuits that are thought to underlie successful retrieval and have also been described in patients with HCV infection (49).

### Other Viral Infections and Cognitive Decline

The role of viral infections, especially from *Herpesviridae* family, as a causal relation of cognitive decline and dementia, has been debated for over a decade (50). Among the plausible mechanisms, a recent infection or the latent HSV1 virus reactivation could unfold a cascade of neuroinflammation, aside from influencing the production of amyloid peptide (Aβ) and tau protein, central findings in the pathophysiological mechanism of AD (51, 52). A recent systematic review and metanalysis has found possible association between varicella-zoster virus (VZV) clinical reactivation, especially in cases of ophthalmic zoster and the increased risk of dementia (53).

Evidence indicates that SARS-CoV-2, with evident neurotropism, may cause unfavorable neurological outcomes, among which, cognitive decline, although no long-term studies have arisen yet (54). Recent publications showed cognitive deficits in subacute phases of COVID-19, such as working memory, attention, naming, processing speed, memory coding, visual and verbal memory, and executive functions (55, 56).

### Summarizing Evidence on Chikungunya Virus Neurovirulence

There are a few data in the literature regarding the cognitive impact of CHIKV infection, and the studies that objectively assessed cognition were performed exclusively on children who were infected in the perinatal period. In the CHIMERE cohort study, 51% of the children who had been infected through vertical transmission presented, at age of 2, deficits in language, coordination, socialization, and posture (15). The study of van Ewjik et al., an uncontrolled prospective cohort, evaluated children that were severely affected by CHIKV in their first 6 months of life, identifying cognitive impairment in 41% of the children, and also at age 2 (16). In contrast, Waechter et al. in a recent cohort study involving 683 children, did not observe significant decline in neurodevelopment at age 2, either being exposed to the virus in the womb or postnatally (17).

Although subjective memory and attention complaints have been reported by telephone interviews in the TELECHIK survey (14), there is no publication, to date, addressing post-infection neuropsychological evaluation in young or older adults. One follow-up study of 21 patients with CHIKV encephalitis in La Reunion island resulted in two cases of cognitive decline/dementia (clinical picture not described), although this figure could be greater once seven people had died and four had lost follow-up (10).

A recent paper revised the neurologic phenomena of the World Health Organization's list of 20 infectious diseases with endemic or pandemic potential, in which chikungunya disease is included (57). Considering the evident increase in life expectancy together with the burden of cognitive and physical disability, especially in low- and middle-income countries (58), appropriate attention should be given to chikungunya long-term adverse outcomes in the oldest ages.

To the best of our knowledge, this is the first study to analyze cognitive impairment in older adults after chikungunya fever. Our data suggest that CHIKV infection may cause long-term cognitive decline in aged people regardless of CNS presentations in acute chikungunya. In our sample, CHIK subjects had more subjective cognitive complaints and objective decline in a global screening test (MoCA) and a trend toward dysfunction in executive functions (cognitive flexibility, inhibitory control), processing speed, semantic and episodic memory. We conclude that CHIKV may cause cognitive impairment in old ages and, therefore, is a risk factor for future dementia in this age group. Along with other CNS involvement in neurotropic virus infections and the neuropsychological profile of our sample, the presented data might suggest that CHIKV is possibly involved in frontal-subcortical network disruption, which are critical to cognitive functioning.

This study has several limitations. Our sample was mainly represented by elders with higher income and education, which does not reflect the Brazilian reality. Such disparity is explained by the geographic location in Natal, where the 2019 chikungunya outbreak arose, an upper and upper-middle class neighborhood. The study in question started just before the COVID-19 pandemics; hence, the evaluation of healthy volunteers was compromised, resulting in an imbalance between controls and subjects previously infected by chikungunya. The aforementioned reason also impaired the follow-up and neuroimaging studies of these patients in order to understand the nature and progression of the cognitive deficit. Finally, the possibility of selection bias must be considered once older adults who perceived more long-term morbidity after the acute disease might have volunteered more.

Chikungunya fever is endemic in many continents and has been recently considered a priority by the World Health Organization. Increased efforts toward vector control and the development of effective therapies are paramount to prevent its long-term burden. Future research is needed to fully understand the pathophysiology of cognitive damage, such as CSF and neuroimaging biomarkers, as well as longitudinal observation of older patients after the acute disease.

## Data Availability Statement

The raw data supporting the conclusions of this article will be made available by the authors, without undue reservation.

## Ethics Statement

The studies involving human participants were reviewed and approved by 20420119.0.0000.5292. The patients/participants provided their written informed consent to participate in this study.

## Author Contributions

VP was main responsible for the conduction of the study, the research interviews, and the writing and editing of the manuscript. JA assisted with the neuropsychological data collection and correction and statistical analysis. KL reviewed the manuscript. KA was responsible for the oversight of the study and commented on and edited the manuscript. All authors contributed to the study design, data interpretation, and gave the final approval for its publication.

## Funding

This study was supported by Coordenação de Aperfeiçoamento de Pessoal de Nível Superior - Brasil (CAPES) - Finance Code 001.

## Conflict of Interest

The authors declare that the research was conducted in the absence of any commercial or financial relationships that could be construed as a potential conflict of interest.

## Publisher's Note

All claims expressed in this article are solely those of the authors and do not necessarily represent those of their affiliated organizations, or those of the publisher, the editors and the reviewers. Any product that may be evaluated in this article, or claim that may be made by its manufacturer, is not guaranteed or endorsed by the publisher.
