# Supplementary material for: Cognitive Dysfunction of Chikungunya Virus Infection in Older Adults
Source: Front Psychiatry. 2022 Apr 5;13:823218. doi: 10.3389/fpsyt.2022.823218 (PMC9016789; doi:10.3389/fpsyt.2022.823218)
Supplement: Supplementary file 1 [file Data_Sheet_1.PDF]

SUPPLEMENTARY MATERIAL

This session brings the exploratory analysis and some aspects of a more technical nature that justified the statistical modeling of the influence of chikungunya infection on the classification by MoCA test while controlling for demographic potentially confounding variables. The results, whose inference is supported on probabilistic grounds, are described in the main test.

Exploratory analysis

At first, the sample distribution of the scores from the MoCA test seems to differ among participants who had been infected with the chikungunya virus as compared to control healthy peers. Accordingly, Figure S1 (A and B) shows the central tendency for these scores that looks displaced toward lower values after chikungunya infection. As a result, the proportion of healthy controls classified as normal in terms of MoCA score (presenting scores higher than 25) seems to be considerably higher than among elders previously infected with chikungunya virus. Because such a binary classification based on threshold holds higher clinical relevance, it became the main metric of MoCA test along this analysis.

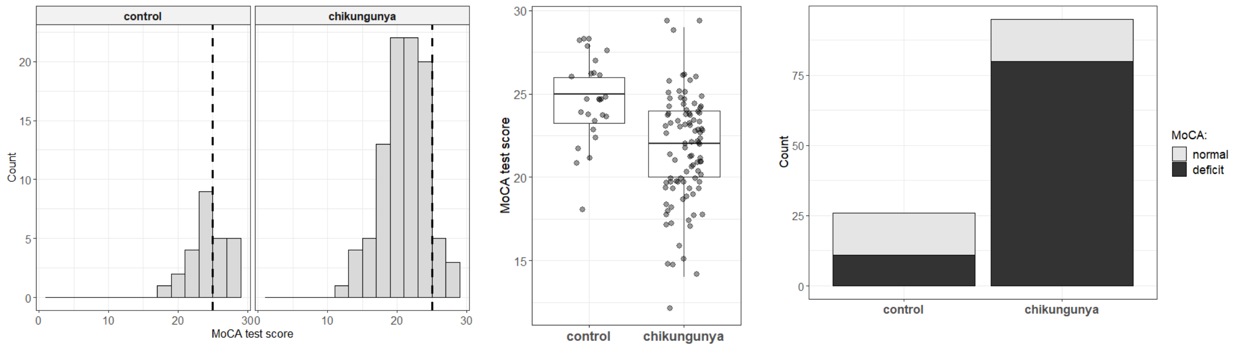

Figure S1: the influence of chikungunya infection on the MoCA test. The sample distribution of MoCA scores (A) are summarized according to quartiles in B. Next, The proportion of participants considered to have a normal performance on such test (whose scores were at least 25) is illustrated according to the occurrence of chikungunya infection.

In turn, the performance on the MoCA test looks insensitive to the genders of the participants. This pattern is apparent both when the scores (Figure S2-A) and the classification (Figure S2-B) are concerned. Yet, gender seems not to modulate the influence of chikungunya infection on MoCA, as this relationship is preserved on both genders (Figure S2-C and Figure S2-D). Thus, for the purpose of explaining the performance on the MoCA test, the gender looks devoid of relevance.

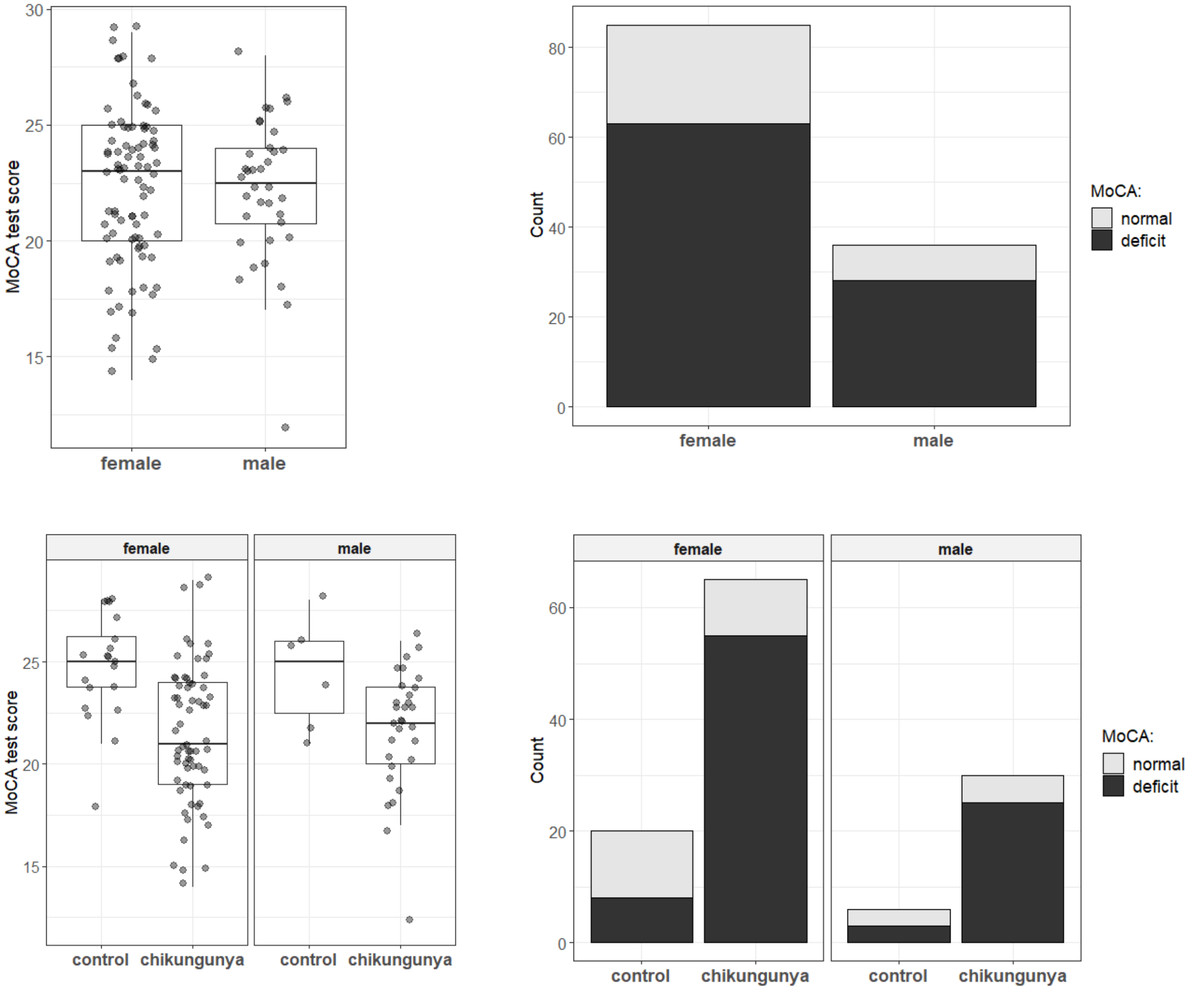

Figure S2: the influence of gender on the performance on the MoCA test. A) sample distribution of test scores is summarized by boxplots according to each gender. B) gender is used to distinguish the proportion of participants, whose performance on the test was classified as normal (score greater or equal to 25). The putative modulation by gender of the relationship between chikungunya infection and MoCA test is addressed in C and D.

In sequence, Figure S3-A portrays a fuzzy relationship between age and the scores from the MoCA test. From the qualitative perspective, no clear pattern stands out, so that the independence between these variables remains reasonable. In addition, such scenario does not seem to change whether the participant had been infected with chikungunya virus or not, indicating that an interaction between the infections and age is rather unexpected. However, a detrimental influence of age looks to emerge when the binary classification of MoCA is taken into account, as the proportion of participants considered deficient increases with growing age (except for the oldest category; Figure S3-B).

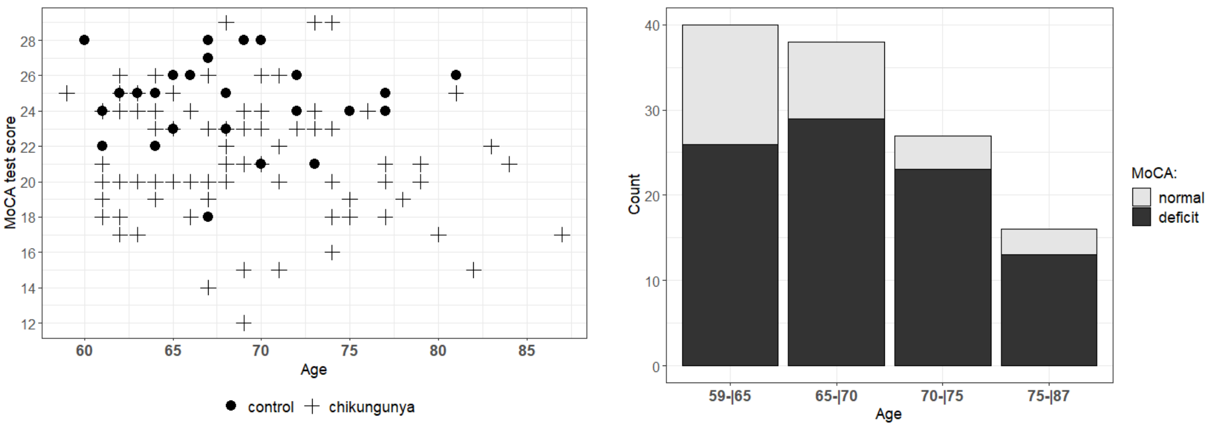

Figure S3: the influence of age on the performance on the MoCA test. A) Age and chikungunya infection are related to the scores from the MoCA test. B) vertical bars illustrate the proportion of participants classified as cognitively impaired in the MoCA test according to age.

In turn, marital status looks irrelevant to the performance on the MoCA test both when the scores (Figure S4-A) and the binary classification (Figure S4-B) are taken into account, being any discrepancy likely to result from reduced sample size for some of the categories of marital status. To increase the balance (in terms of sample size), the categories other than “married” were grouped as “unmarried”. However, the context looked much the same after grouping the data and the marital status seemed not to affect the relationship between chikungunya infection and the scores of the test (Figure S4-C). In addition, a similar change in the proportion of participants classified as impaired on MoCA as following chikungunya was observed in both married and unmarried groups (Figure S4-D). Altogether, these plots indicate that a direct or indirect effect of marital status on the performance on the MoCA test are unexpected.

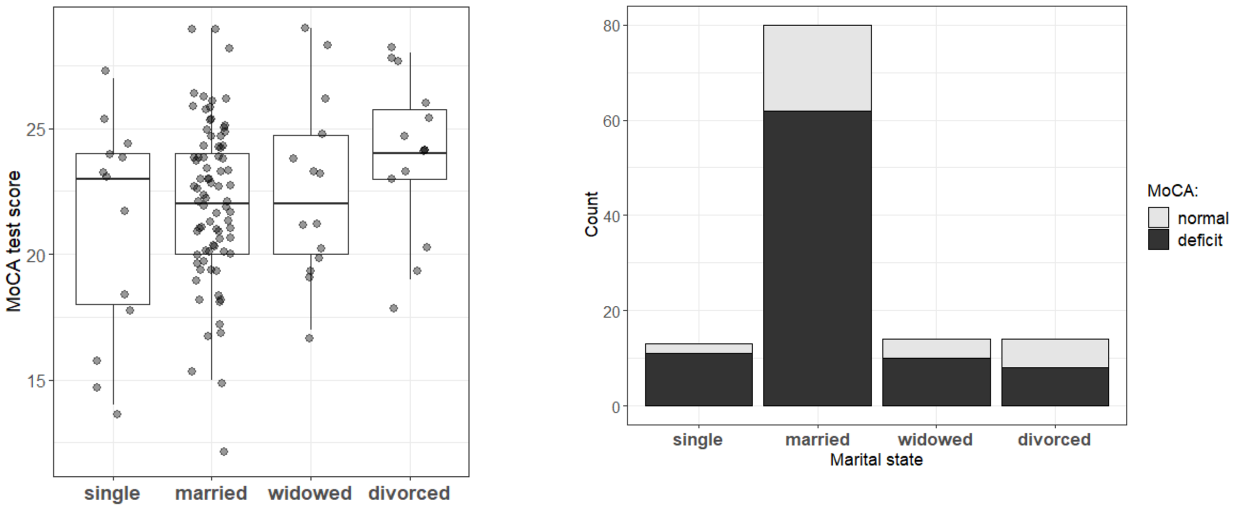

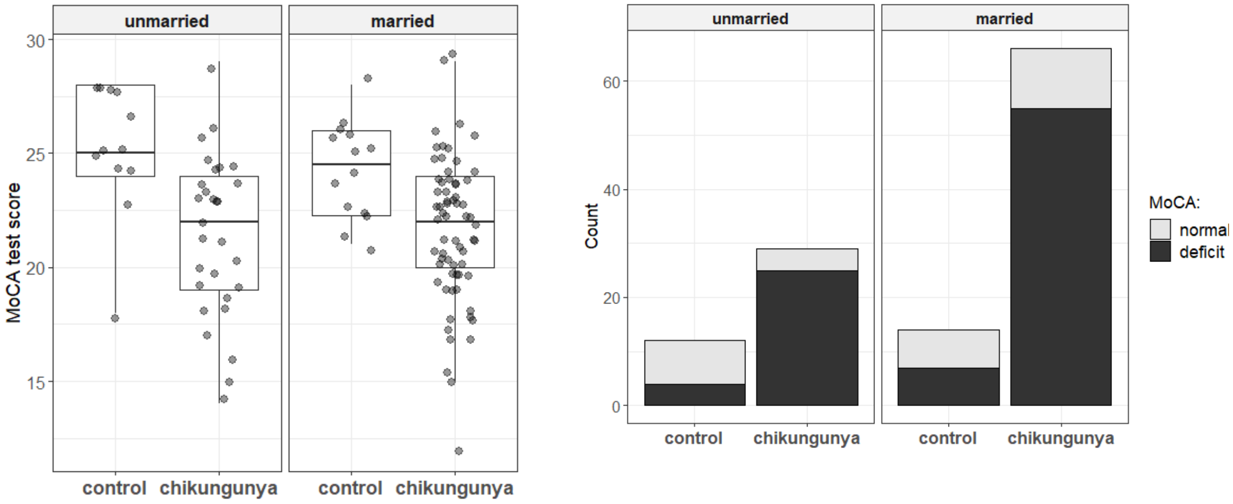

Figure S4: the influence of marital status on cognitive performance as addressed by the MoCA test. The relationship between the marital status and the MoCA is summarized regarding the scores of the test and corresponding binary classification in A and B, respectively. For C and D, categories other than "married" were grouped as "unmarried" and used to further illustrate a putative influence on the relationship between chikungunya infection and MoCA performance.

In addition, the MoCA test seems also to be insensitive to the years of schooling both when the ordinal scores are considered (Figure S5-A) and when the binary classification of the performance on the test is taken into account (Figure S5-B). Moreover, years of schooling looks to spare the effect of chikungunya infection on MoCA from any influence, since the relationship of the performance on such test with previous contagion with the virus remains stable among the levels of education (Figure S5-C and Figure S5-D). Thus, at least under this qualitative perspective, the sample seems to lack evidence endorsing a substantial influence of the schooling on the cognitive performance on MoCA.

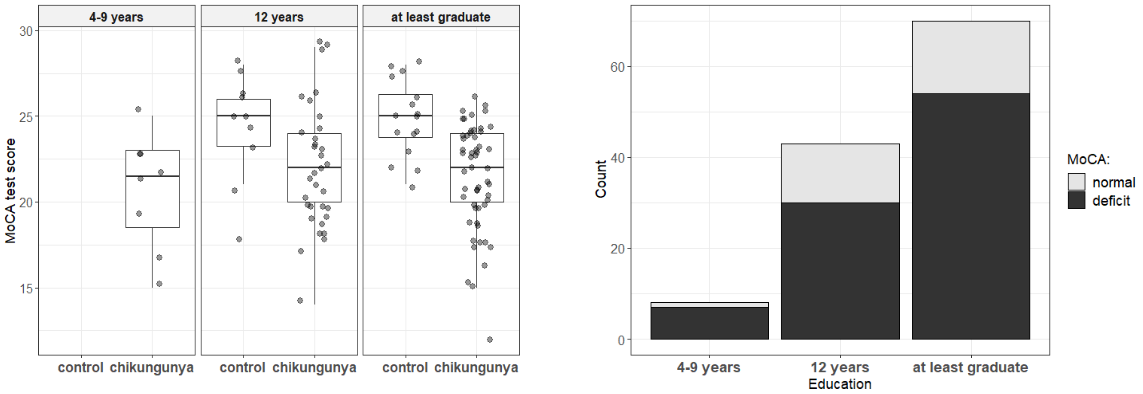

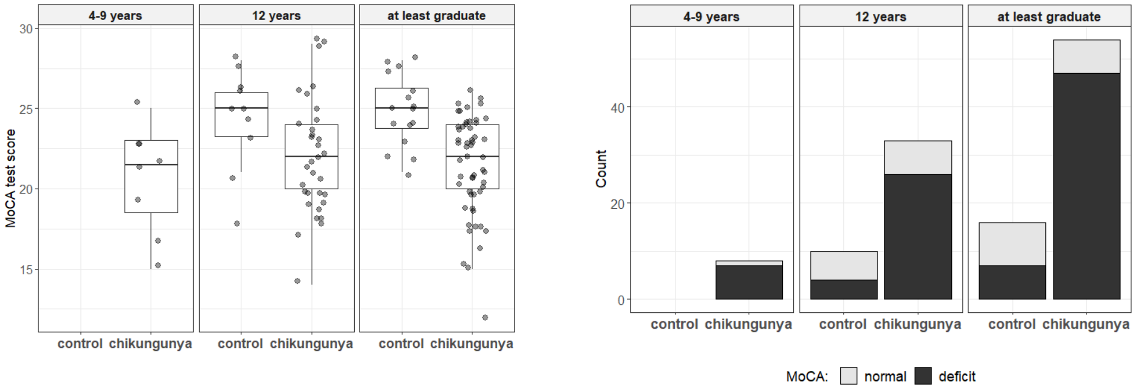

Figure S5: the putative influence of the years of schooling on the cognitive performance on the MoCA test. The relationship of education and MoCA is summarized in A and B for the ordinal scores of the test and its corresponding binary classification, respectively. In C and D, the contrast on MoCA performance between subjects previously infected with chikungunya and healthy controls is presented according to the category of participants' education.

For the appreciation of the relationship between occupation and MoCA performance, unemployed and retired conditions were pooled as the "inactive" category. In this context, Figure S6-A exhibits a broader dispersion of MoCA scores towards lower ranks of the scale among inactive participants as compared to working peers. Accordingly, the proportion of inactive participants classified as deficient in the MoCA test looked substantially higher than among those who were working (Figure S6-B). Despite such a contrast, at least when the classification from the MoCA test was concerned, the occupancy seemed to lack an influence on the effect of chikungunya infection on MoCA, once more subjects classified as deficient in the MoCA had been infected with chikungunya regardless of working or not. In turn, when MoCA scores are taken in account (in ordinal scale), a putative interaction between occupation and chikungunya infection becomes less elusive, although this suspicion may arise from differences in the sample size. At last, under a qualitative perspective, it sounds reasonable to speculate that working categories may be related to participants' age, once retirement tends to occur on older people and unemployment is considerably rarer in this sample.

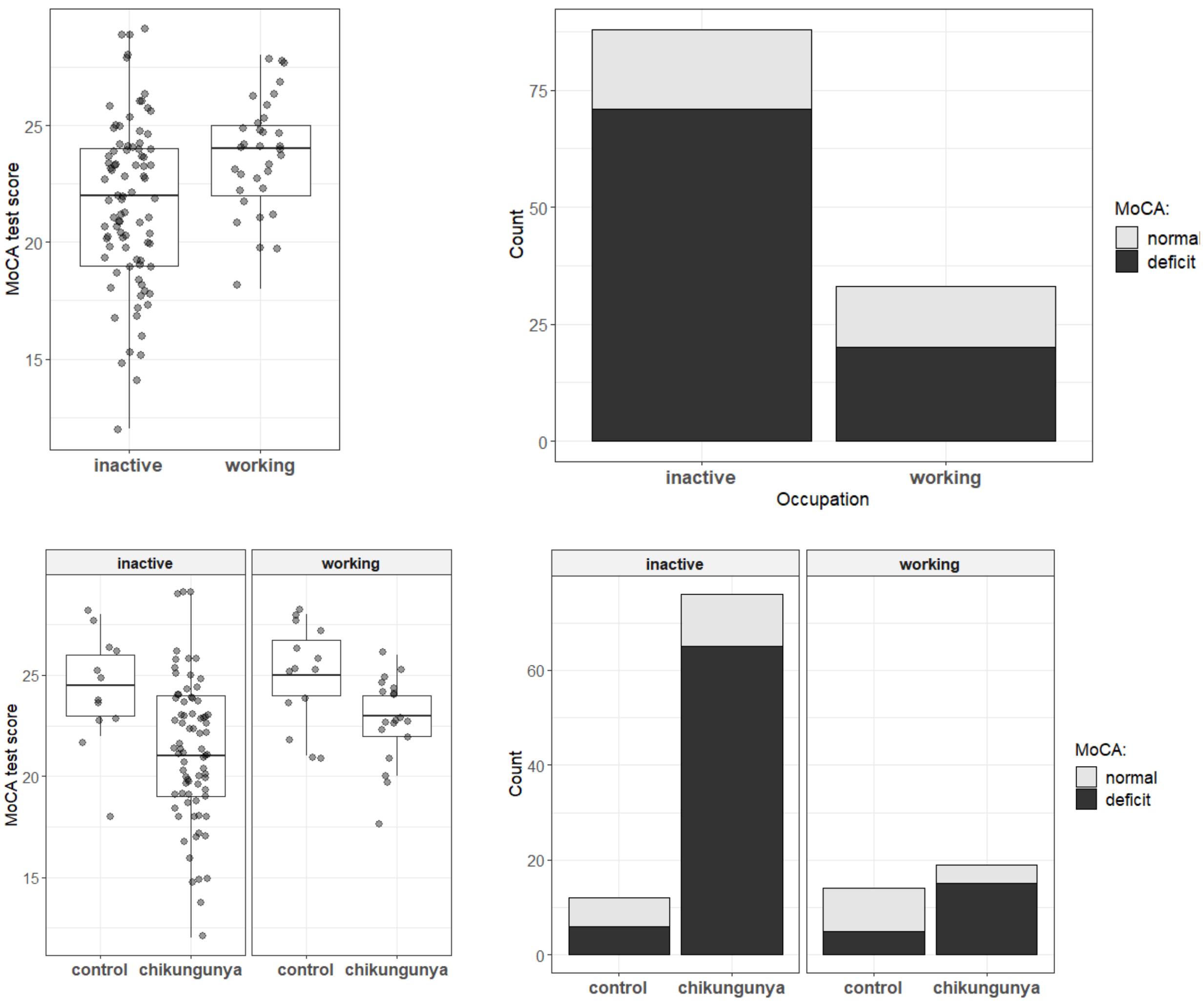

Figure S6: the influence of occupation on the performance on the MoCA test. A) sample distribution of test scores is summarized by boxplots according to whether the participant was working or not. B) occupation is used to distinguish the proportion of participants, whose performance on the test was classified as normal (score greater or equal to 25). At last, the putative modulation by occupation of the relationship between chikungunya infection and MoCA test is addressed in C and D.

At last, Figure S7 illustrates the putative influence of income on cognitive performance on MoCA. In this context, MoCA looks to be insensitive to income, both regarding the ordinal scores (Figure S7-A) and the binary classification on such test (Figure S7-B). Moreover, Figure S7-C and Figure S7-D do not provide appreciable evidence that the income could modulate the effect of chikungunya infection on MoCA performance. Despite some conditions actually counted on with very short sample size, the general pattern representing the difference on the MoCA test between chikungunya infected subjects and healthy control looked to remain constant regardless of the income level.

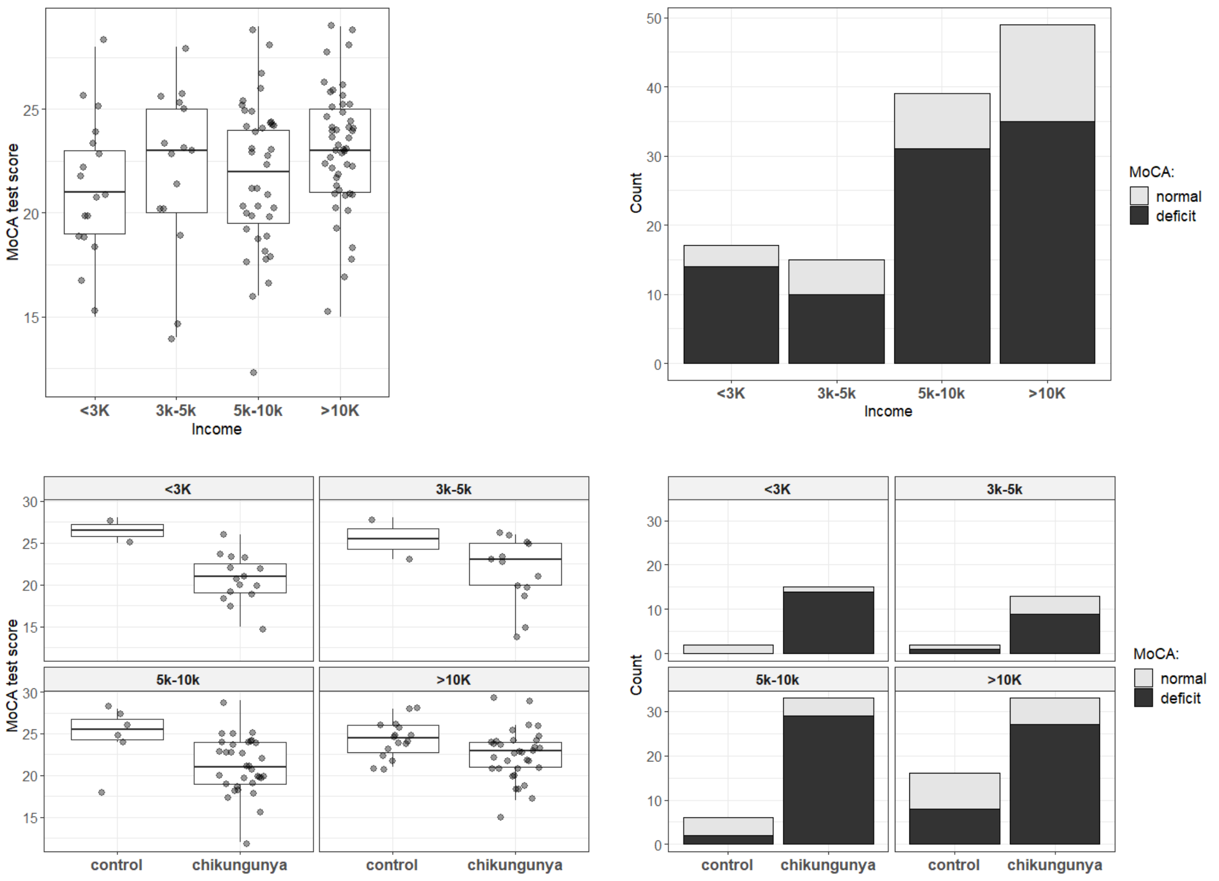

Figure S7: the putative influence of income on cognitive performance on the MoCA test. The relationship between income and MoCA is summarized in A and B for the ordinal scores of the test and its corresponding binary classification, respectively. In C and D, the contrast on MoCA performance between subjects previously infected with chikungunya and healthy controls is presented according to the category of participants' income. Income was measured and is exhibited as Brazilian current currency (reais - R$).

After the graphical inspection, the relationship between predictors and the response (the performance on MoCA) was quantified in terms of polychoric correlation and statistical distance, yet such analysis was expanded to every pair of variables. Except for age, all variables are categorical in nature, thus they were considered as grouped versions to mitigate sparsity. As expected, chikungunya presented higher correlation and shorter distance to the binary classification of the MoCA test. Beyond CHIKV, MoCA looks somewhat related to age and occupation. However, occupation was found to be considerably correlated to CHIKV, so that both variables should not be included in the same statistical model as predictors in order to avoid that multicollinearity undermined the precision on estimation based on such a model. Because chikungunya infection is central to scopus of the present study (and it also looks to have higher influence on MoCA test), CHIKV turned out to be preferable to occupation to embody the statistical model. Similarly, occupation seems also to hold considerable correspondence to age. But, correlation between age and CHIKV looks much less discouraging (and reasonably explained by the chance) so that their presence as predictors in the same model is expected to spare estimation from multicollinearity.

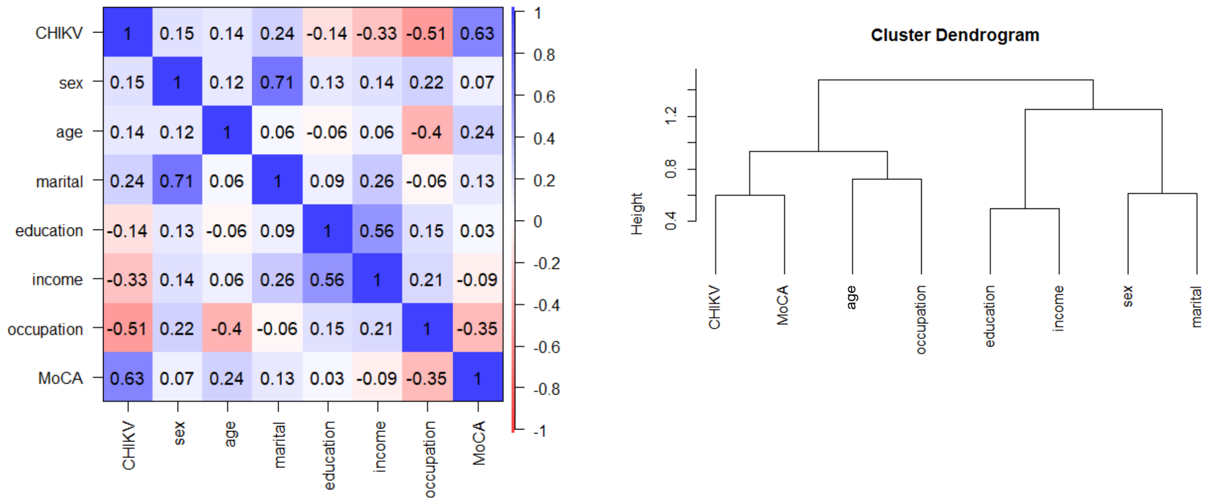

Figure S8: the quantification of the degree of correspondence among the pairs of variables. A) the relationship between each pair of variables is quantified in terms of polychoric correlation. B) a dendrogram illustrates the distance as a measure of dissimilarity among the variables. In this context, the shorter the height connecting variables, the more closely related are the variables.

Diagnostic measures

From the exploratory analysis, the infection with chikungunya and age stand out as the most promising predictors to explain the probability of having the performance on the MoCA test classified as deficient. Such a configuration for the model was corroborated by the backward stepwise algorithm of automatic selection of predictors. In addition, some related options were manually checked, but discarded.

Next, the elected model was evaluated according to its diagnostic measures. Figure S9-A indicates that a single observation (identified by corresponding index in the dataset, #9) stands out regarding leverage. Despite being visually detached from remaining points, the absolute difference on the magnitude of h is considerably small, hence of minor importance. Under this context, it sounds reasonable to assume that all observations count with similar amounts of information to be estimated by the model. The same point is also highlighted according to its influence on the estimation of the parameters of the model (Figure S9-B), although such an influence is overweld by another observation (#88). This means that the estimation of the parameters of the model is much more influenced by these individual points than by any other single observation. In other words, these influential points introduce some bias in the model towards the condition they represent. Even though, the statistical model was efficient in modeling (i.e., predicting) every observation (Figure S9-C), so that given the model no observation turned out to be weird. Altogether, these analyses indicate that the model works properly, although the bias introduced by the influential points deserves further scrutiny.

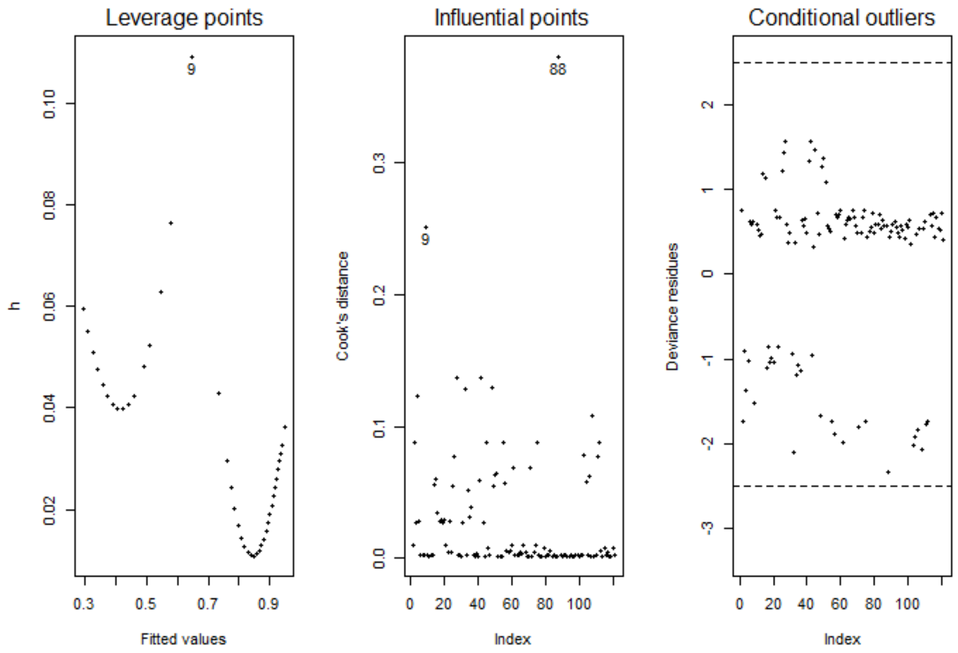

Figure S9: diagnostic measure of the logistic regression model to explain the probability of impaired performance on the MoCA test according to the occurrence (or not) of chikungunya infection and participant's age.

Confirmatory analysis

The bias introduced by the influential observations was addressed by means of the comparison between the original estimates of the model's parameters and corresponding p-value (on Wald test) and those statistics generated after a specific participant was dropped from the sample. Under this circumstance, removing any of the influential observations caused a minor decrease (0.4%) in the coefficient modeling the effect of chikungunya infection on MoCA, a deflection that did not appreciably modify the corresponding p-value (Table S1). Thus, the bias introduced by influential observations on the effect of the infection is negligible. In turn, any of the influential points were actually mitigating the effect of age on MoCA, once dropping any of these participants results in a 71.4% increment in the coefficient addressing the effect of age. As a result, the corresponding p-value turns out to be statistically significant and the inclusion of age in the model as a predictor becomes unquestionable. Both influential participants were 81 years old (the only ones with this age) and they were classified as normal for the MoCA test. In addition to them, only one subject out of 20 among the individuals older than 74 were classified as normal and no other one older than 77 years received such a grade. In other words, they seemed unusual to the statistical model because their performance was exceptionally normal considering their age. As a result, any of these observations precluded the statistical significance of the effect of age on the MoCA test classification. In conclusion, the general pattern of the data endorses that the probability of having the MoCA performance classified as impaired increases as the subject grows old, although, at a first glance, such pattern remains hindered by the bias introduced by only two unusually influential observations.

Confirmatory analysis

Coefficients	Estimates			p-values		
	original	delta 9	delta 88	original	9	88
(Intercept)	-5.063	-0.645	-0.645	0.089	0.016	0.016
Chikungunya	1.956	0.004	0.004	0.000	0.000	0.000
Age	0.070	-0.714	-0.714	0.106	0.019	0.019

Table S1: confirmatory analysis for the statistical model (introduced in Table 1) fitted to explain the influence of chikungunya infection and age on the probability of having the performance on MoCA test classified as deficient. The influential observations were defined according to corresponding Cook's distance (illustrated in Figure S9-B). Under "Estimates" head, "delta" indicates a proportional decrease in the original estimate of the corresponding coefficient. At last, the number labeled in each column denotes the index of the influential observation that was dropped.

Envelope

At last, Figure S10 illustrates the expected behavior of the residues given the assumption of the model. Once residues represent the probabilistic component of the statistical model and they are all constrained within the confidence band of the envelope, we can safely conclude that assumptions of the model are in compliance with the data. Such correspondence validates the model, hence the conclusions deriving from it.

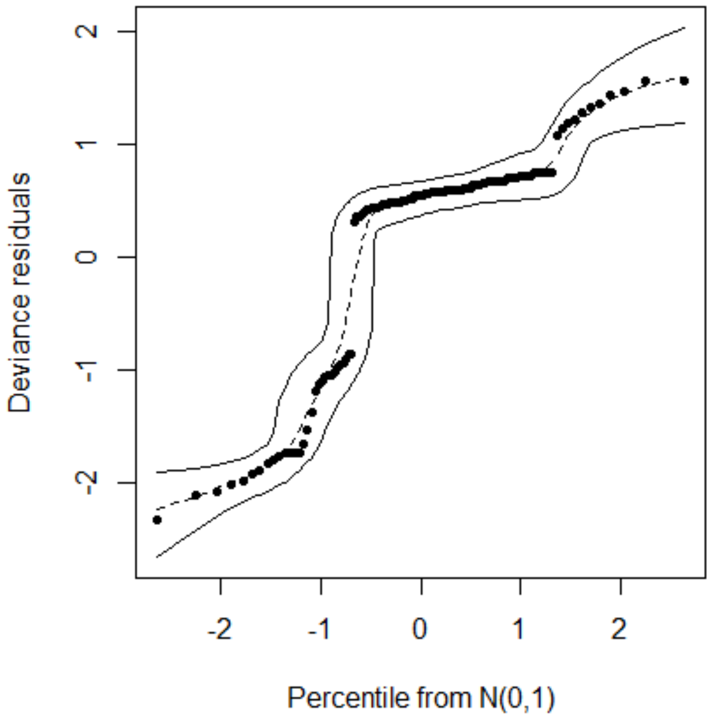

Figure S10: the envelope illustrating the confidence band for the residues given the assumptions of the statistical model.
